# Di-μ-acetato-κ^4^ *O*:*O*′-μ-oxido-κ^2^ *O*:*O*′-bis­[*cis*-(2,2′-bipyridine-κ^2^ *N*,*N*′)-*trans*-(pyridine-κ*N*)ruthenium(III)] bis­(hexa­fluoridophosphate)

**DOI:** 10.1107/S1600536813003334

**Published:** 2013-02-09

**Authors:** Yohei Ido, Takashi Fujihara, Akira Nagasawa

**Affiliations:** a Department of Chemistry, Graduate School of Science and Engineering, Saitama University, Shimo-Okubo 255, Sakura-ku, Saitama 338-8570, Japan; b Comprehensive Analysis Center for Science, Saitama University, Shimo-Okubo 255, Sakura-ku, Saitama 338-8570, Japan

## Abstract

The hemerythrin-type dinuclear title complex, [Ru_2_(CH_3_COO)_2_O(C_10_H_8_N_2_)_2_(C_5_H_5_N)_2_](PF_6_)_2_, consists of two Ru^III^ ions with a six-coordinate octa­hedral geometry, bridged by an oxide and two acetate ligands, with a bidentate 2,2′-bipyridine ligand and a pyridine ligand bonding at terminal positions. The Ru—Ru distance and Ru—O—Ru angle are 3.2838 (3) Å and 121.79 (7)°, respectively, and the average Ru—N(pyridine) bond length is 2.164 (8) Å. Several C—H⋯F, C—H⋯O and C—H⋯N inter­actions generate a three-dimensional network in the crystal structure. π–π stacking inter­actions [centroid–centroid distance = 3.6389 (3) Å] between inversion-related 2,2′-bipyridine rings are also observed.

## Related literature

For related structures, see: Zhang *et al.* (2011 ▶); Sudha & Chakravarty (1996 ▶). For background to hemerythrin-type diruthenium(III) complexes, see: Abe *et al.* (2002 ▶); Dean (1985 ▶); Tembe & Ganeshpure (1999 ▶); Fukumoto *et al.* (1998 ▶); Inomata *et al.* (1999 ▶); Sasaki (1995 ▶); Sasaki *et al.* (1991 ▶); Valli *et al.* (1997 ▶). For the synthesis, see: Sasaki *et al.* (1991 ▶); Ido *et al.* (2013 ▶).
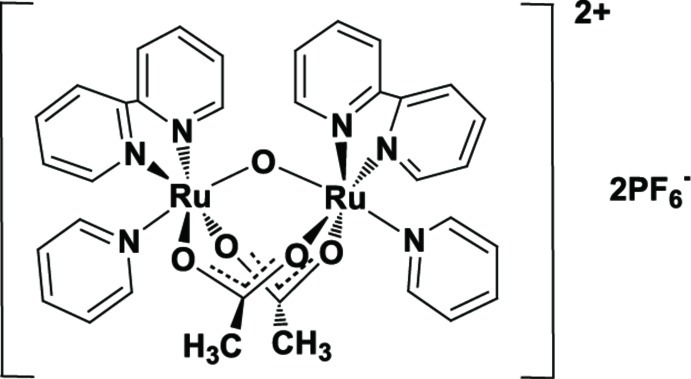


## Experimental

### 

#### Crystal data

[Ru_2_(C_2_H_3_O_2_)_2_O(C_10_H_8_N_2_)_2_(C_5_H_5_N)_2_](PF_6_)_2_
*M* *_r_* = 1096.74
Monoclinic, 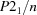

*a* = 12.3330 (9) Å
*b* = 18.2182 (14) Å
*c* = 18.1490 (14) Å
β = 97.253 (1)°
*V* = 4045.2 (5) Å^3^
*Z* = 4
Mo *K*α radiation
μ = 0.93 mm^−1^
*T* = 150 K
0.16 × 0.14 × 0.08 mm

#### Data collection

Bruker APEXII CCD area-detector diffractometer
Absorption correction: multi-scan (*SADABS*; Bruker, 2008 ▶) *T*_min_ = 0.865, *T*_max_ = 0.929
21514 measured reflections
8544 independent reflections
7343 reflections with *I* > 2σ(*I*)
*R*_int_ = 0.039

#### Refinement

*R*[*F* ^2^ > 2σ(*F* ^2^)] = 0.024
*wR*(*F* ^2^) = 0.063
*S* = 1.02
8544 reflections
552 parameters
H-atom parameters constrained
Δρ_max_ = 0.47 e Å^−3^
Δρ_min_ = −0.69 e Å^−3^

### 

Data collection: *APEX2* (Bruker, 2008 ▶); cell refinement: *SAINT* (Bruker, 2008 ▶); data reduction: *SAINT* and *XPREP* (Bruker, 2008 ▶); program(s) used to solve structure: *SHELXS97* (Sheldrick, 2008 ▶); program(s) used to refine structure: *SHELXL97* (Sheldrick, 2008 ▶); molecular graphics: *ORTEP-3 for Windows* (Farrugia, 2012 ▶); software used to prepare material for publication: *XCIF* (Bruker, 2008 ▶).

## Supplementary Material

Crystal structure: contains datablock(s) global, I. DOI: 10.1107/S1600536813003334/gg2109sup1.cif

Structure factors: contains datablock(s) I. DOI: 10.1107/S1600536813003334/gg2109Isup2.hkl

Additional supplementary materials:  crystallographic information; 3D view; checkCIF report

## Figures and Tables

**Table 1:** Hydrogen-bond geometry (Å, °)

*D*—H⋯*A*	*D*—H	H⋯*A*	*D*⋯*A*	*D*—H⋯*A*
C9—H9⋯F2	0.95	2.53	3.227 (3)	130
C10—H10⋯F5	0.95	2.48	3.362 (3)	154
C10—H10⋯O4	0.95	2.59	3.105 (3)	115
C12—H12⋯F6^i^	0.95	2.42	3.270 (3)	149
C15—H15⋯O4	0.95	2.43	2.904 (3)	110
C17—H17*A*⋯F1^ii^	0.98	2.34	3.240 (3)	153
C18—H18⋯F12	0.95	2.37	3.090 (3)	132
C18—H18⋯O3	0.95	2.52	3.096 (3)	119
C24—H24⋯O1^iii^	0.95	2.56	3.405 (3)	149
C27—H27⋯F5	0.95	2.32	3.101 (3)	139
C28—H28⋯O3	0.95	2.23	2.855 (3)	122
C32—H32⋯N5	0.95	2.49	3.080 (3)	121
C34—H34*C*⋯F7^iv^	0.98	2.52	3.455 (3)	160

Symmetry codes: (i) 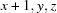; (ii) 
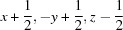
; (iii) 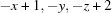; (iv) 
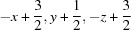
.

**Fig. 1.**
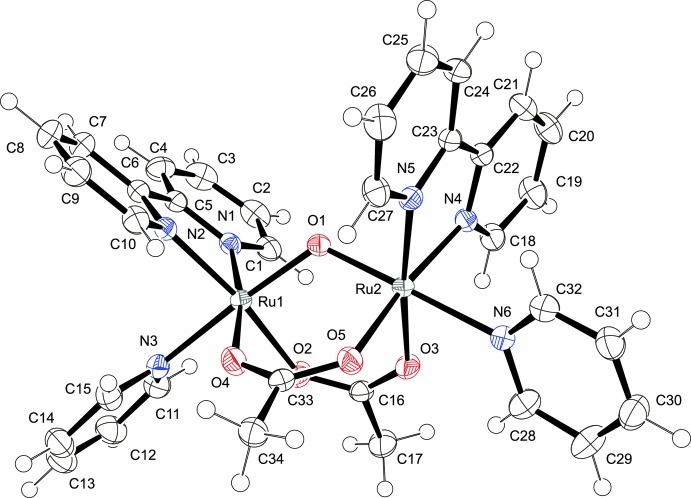
The molecular structure of the complex cation of (I). Displacement ellipsoids are drawn at the 50% probability level.

**Fig. 2.**
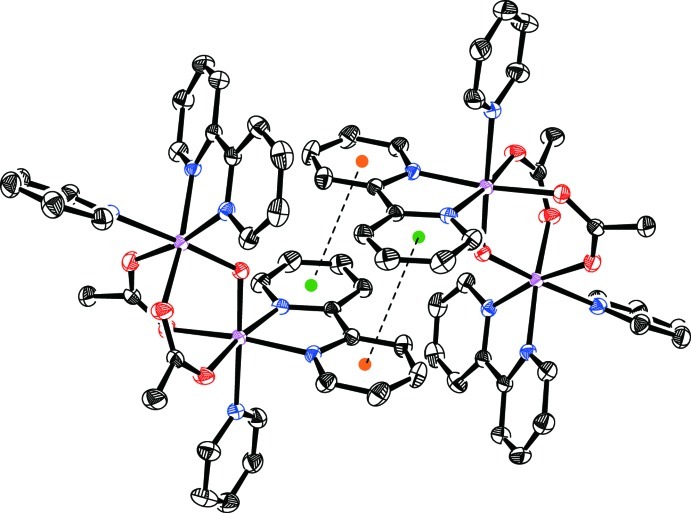
The complex cations are linked by π- π interactions between neighbouring 2,2'-bipyridine ligands with centroid-centroid distance of 3.6389 (3) Å.

## supplementary crystallographic information

### Comment

Several hemerythrin type diruthenium(III) complexes have been studied. These complexes are characterized by the unique core which consists of two Ru^III^ ions and a *µ*-oxido ligand and two carboxylato ligands. For example, the Ru–Ru distance and Ru–O–Ru angle of the complex with pyridine at *cis-to-oxido* position, [Ru^III^_2_(CH_3_CO_2_)_2_O(C_5_H_5_N)_6_](PF_6_)_2_ (III), are 3.251 (2) Å and 122.2 (5)°, respectively, (Sasaki *et al.* 1991). Although the substitution, redox and spectroscopic properties of the title complex have been reported, the structure in the solid state remains unexplored. We report here the determination of the structure of I. The molecule has a hemerythrin type diruthenium(III) core {Ru^III^_2_(CH_3_CO_2_)_2_O}^2+^ which consists of two Ru^III^ ions in a six-coordinated octahedral geometry and terminal ligands (2,2'-bipyridine and pyridine). The Ru–Ru distance and the Ru–O–Ru angle are 3.2838 (3) Å and 121.79 (7)°, respectively. The average of Ru–N bond lengths at the *trans-* and at the *cis-*sites to the bridging oxido are 2.1635 and 2.0292 Å, respectively. The former is longer than the latter, and this could be interpreted as in order to the *trans* influence of the *µ*-oxido, which is a stronger electron donating ligand than acetato oxygen atoms. The average Ru–N*_trans_* length is shorter than that of III (2.185 Å), and longer than that of the complex with 1-methylimidazole instead of pyridine at the *trans*-*to*-*oxido* position, [Ru^III^_2_(CH_3_CO_2_)_2_O(C_10_H_8_N_2_)_2_(C_4_H_6_N_2_)_2_](PF_6_)_2_ (IV) (2.125 Å) (Sudha & Chakravarty, 1996). The former may be due to the steric effect of the ligands at the *cis-to-oxido* position: the molecular plane of the flat 2,2'-bipyridine in I is coplanar with the plane consisting of four *cis-to-oxido* positions ("*cis* plane"), and does not hinder the bonding of pyridine at the *trans* position, while two pyridine molecules at the *cis-to-oxido* position in II are almost perpendicular to the *cis* plane, and some steric interactions may be possible. The electronic effect is probable for the latter case: pyridine is weaker Lewis base (protonation constant exponent p*K*_a _= 5.17; Dean, 1985) than 1-methylimidazole is (p*K*_a _= 7.06; Dean, 1985), and Ru–N*_trans_*(pyridine) in I bond is weaker than Ru–N*_trans_*(1-methylimidazole) in IV. In addition, steric interactions of vicinal protons of N*_trans_* with 2,2'-bipyridine on the *cis* plane are stronger with 2- and 6-protons on the six-membered ring of pyridine in I than with the 2- and 5-protons on the five-membered ring of 1-methylimidazole in IV, with the result that they gives more negative effect on bonding in I. On the other hand, though the average length of Ru–N*_cis_*(2,2'-bipyridine) in I (2.0292 Å) is similar to that in IV (2.030 Å), these lengths are shorter than that of Ru–N*_trans_*(pyridine) in III (2.087 Å). This fact shows that Ru–N*_cis_* distance is influenced by the steric hindrance of ligands at the *cis* position rather than the electronic effect of ligands at the *trans* (pyridine and 1-methylimidazole) or *cis* (pyridine and 2,2'-bipyridine(p*K*_a_ = 4.35; Dean, 1985)) sites. In the crystal structure the cations and anions are linked by C-H···F, C-H···O and C-H···N hydrogen bond interactions. In addition, π-π stacking interactions between neighbouring 2,2'-bipyridine ligands are also observed with a shortest centroid-centroid distance of 3.6389 (3) Å (Fig. 2; [*Cg*1···*Cg*2 (1 - *x*, - *y*, 2 - *z*) and *Cg*2···*Cg*1 (1 - *x*, - *y*, 2 - *z*)] where *Cg*1 and *Cg*2 are the N4/C18–C21 and N5/C23–C27 rings, respectively).

### Experimental

The complex was synthesized previously (Sasaki *et al.* 1991), but we have prepared single crystals by another method. The complex with nitrile-*κN*s at the both *trans-to-oxido* sites, [Ru^III^_2_(CH_3_CO_2_)_2_O(C_10_H_8_N_2_)_2_(C_2_H_3_N)_2_](PF_6_)_2_ (II) (10 mg, 1.0 × 10 ^-5^ mol; Ido *et al.*, 2013), was dissolved in CH_3_CN. Pyridine (82 mg, 1.0 × 10 ^-3^ mol) was dissolved in this solution and kept for 12 h at 333 K. The solution was dried under vacuum to obtain precipitates, which were then recrystallized from the solution in CH_3_CN by adding Et_2_O, and washed with Et_2_O. A blue crystalline product was obtained in 73% yield. By evaporating a concentrated solution in CD_3_CN, single crystals of I were obtained. ^1^H NMR (in CD_3_CN, 500 MHz, 300 K): *δ* 8.66 (4*H*, pyridine), 8.54 (4*H*, 2,2'-bipyridine), 8.12 (4*H*, pyridine), 7.8 (8*H*, pyridine and 2,2'-bipyridine), 7.24 (4*H*, 2,2'-bipyridine), 6.07 (4*H*, 2,2'-bipyridine), 2.09 (6*H*, CH_3_). UV/Vis (CH_3_CN): *λ*_max_/nm (*ε*/M^-1^ cm^-1^) = 599 (19600), 463 (4500), 340 (sh), 285 (43400), and 242 (28500). Elemental analysis: found (calculated): C 36.79 (37.24), H 2.89 (2.94), N 7.55 (7.66)%.

### Refinement

The H atoms were placed in calculated positions, with C—H = 0.95 Å , and refined using a riding model, with *U*_iso_(H) = 1.2*U*_eq_ for aromatic H atoms or 1.5*U*_eq_ for methyl ones. Solvent accessible voids of 37Å^-3^ are present in the lattice.

### Crystal data

**Table:**

[Ru_2_(C_2_H_3_O_2_)_2_O(C_10_H_8_N_2_)_2_(C_5_H_5_N)_2_](PF_6_)_2_	*F*(000) = 2176
*M**_r_* = 1096.74	*D*_x_ = 1.801 Mg m^−^^3^
Monoclinic, *P*2_1_/*n*	Mo *K*α radiation, λ = 0.71073 Å
Hall symbol: -P 2yn	Cell parameters from 9917 reflections
*a* = 12.3330 (9) Å	θ = 2.2–28.3°
*b* = 18.2182 (14) Å	µ = 0.93 mm^−^^1^
*c* = 18.1490 (14) Å	*T* = 150 K
β = 97.253 (1)°	Block, violet
*V* = 4045.2 (5) Å^3^	0.16 × 0.14 × 0.08 mm
*Z* = 4	

### Data collection

**Table:**

Bruker APEXII CCD area-detector diffractometer	8544 independent reflections
Radiation source: Bruker TXS fine-focus rotating anode	7343 reflections with *I* > 2σ(*I*)
Bruker Helios multilayer confocal mirror monochromator	*R*_int_ = 0.039
Detector resolution: 8.333 pixels mm^-1^	θ_max_ = 26.7°, θ_min_ = 1.6°
φ and ω scans	*h* = −15→15
Absorption correction: multi-scan (*SADABS*; Bruker, 2008)	*k* = −23→18
*T*_min_ = 0.865, *T*_max_ = 0.929	*l* = −22→19
21514 measured reflections	

### Refinement

**Table:**

Refinement on *F*^2^	Primary atom site location: structure-invariant direct methods
Least-squares matrix: full	Secondary atom site location: difference Fourier map
*R*[*F*^2^ > 2σ(*F*^2^)] = 0.024	Hydrogen site location: inferred from neighbouring sites
*wR*(*F*^2^) = 0.063	H-atom parameters constrained
*S* = 1.02	*w* = 1/[σ^2^(*F*_o_^2^) + (0.0308*P*)^2^ + 1.0615*P*] where *P* = (*F*_o_^2^ + 2*F*_c_^2^)/3
8544 reflections	(Δ/σ)_max_ < 0.001
552 parameters	Δρ_max_ = 0.47 e Å^−^^3^
0 restraints	Δρ_min_ = −0.69 e Å^−^^3^

### Special details

**Table:**

Geometry. All esds (except the esd in the dihedral angle between two l.s. planes) are estimated using the full covariance matrix. The cell esds are taken into account individually in the estimation of esds in distances, angles and torsion angles; correlations between esds in cell parameters are only used when they are defined by crystal symmetry. An approximate (isotropic) treatment of cell esds is used for estimating esds involving l.s. planes.
Refinement. Refinement of F^2^ against ALL reflections. The weighted R-factor wR and goodness of fit S are based on F^2^, conventional R-factors R are based on F, with F set to zero for negative F^2^. The threshold expression of F^2^ > 2sigma(F^2^) is used only for calculating R-factors(gt) etc. and is not relevant to the choice of reflections for refinement. R-factors based on F^2^ are statistically about twice as large as those based on F, and R- factors based on ALL data will be even larger.

### Fractional atomic coordinates and isotropic or equivalent isotropic displacement parameters (Å^2^)

**Table:**

	*x*	*y*	*z*	*U*_iso_*/*U*_eq_	
C1	0.84969 (17)	0.16178 (12)	0.95696 (13)	0.0266 (5)	
H1	0.8420	0.1450	0.9070	0.032*	
C2	0.94008 (18)	0.14115 (13)	1.00407 (14)	0.0330 (5)	
H2	0.9920	0.1084	0.9877	0.040*	
C3	0.95489 (19)	0.16843 (14)	1.07542 (14)	0.0352 (6)	
H3	1.0192	0.1569	1.1079	0.042*	
C4	0.87585 (18)	0.21250 (13)	1.09933 (13)	0.0309 (5)	
H4	0.8851	0.2319	1.1483	0.037*	
C5	0.78242 (17)	0.22835 (11)	1.05108 (11)	0.0222 (4)	
C6	0.68668 (17)	0.26654 (11)	1.07217 (11)	0.0216 (4)	
C7	0.67667 (19)	0.29137 (12)	1.14324 (12)	0.0284 (5)	
H7	0.7364	0.2870	1.1815	0.034*	
C8	0.5805 (2)	0.32224 (13)	1.15816 (13)	0.0330 (5)	
H8	0.5730	0.3394	1.2067	0.040*	
C9	0.49437 (19)	0.32815 (12)	1.10180 (13)	0.0310 (5)	
H9	0.4267	0.3488	1.1112	0.037*	
C10	0.50830 (17)	0.30350 (11)	1.03152 (12)	0.0252 (4)	
H10	0.4494	0.3081	0.9927	0.030*	
C11	0.82524 (19)	0.35124 (13)	0.89702 (12)	0.0304 (5)	
H11	0.8655	0.3069	0.8964	0.037*	
C12	0.8776 (2)	0.41694 (14)	0.88632 (14)	0.0368 (6)	
H12	0.9523	0.4174	0.8786	0.044*	
C13	0.8198 (2)	0.48151 (14)	0.88702 (14)	0.0387 (6)	
H13	0.8539	0.5273	0.8801	0.046*	
C14	0.7113 (2)	0.47823 (13)	0.89792 (14)	0.0356 (6)	
H14	0.6692	0.5219	0.8984	0.043*	
C15	0.66473 (19)	0.41103 (12)	0.90809 (13)	0.0298 (5)	
H15	0.5898	0.4093	0.9153	0.036*	
C16	0.68155 (17)	0.18333 (11)	0.76443 (11)	0.0225 (4)	
C17	0.7534 (2)	0.18554 (14)	0.70400 (13)	0.0353 (6)	
H17A	0.7181	0.2148	0.6624	0.053*	
H17B	0.7653	0.1355	0.6869	0.053*	
H17C	0.8238	0.2077	0.7230	0.053*	
C18	0.62599 (17)	−0.01977 (12)	0.82185 (12)	0.0269 (5)	
H18	0.6650	0.0083	0.7898	0.032*	
C19	0.65819 (19)	−0.09121 (13)	0.83825 (14)	0.0338 (5)	
H19	0.7182	−0.1120	0.8176	0.041*	
C20	0.6015 (2)	−0.13190 (12)	0.88527 (14)	0.0347 (5)	
H20	0.6227	−0.1810	0.8976	0.042*	
C21	0.51407 (18)	−0.10063 (12)	0.91407 (13)	0.0289 (5)	
H21	0.4747	−0.1278	0.9466	0.035*	
C22	0.48426 (16)	−0.02916 (11)	0.89494 (11)	0.0208 (4)	
C23	0.39162 (16)	0.00954 (11)	0.91996 (11)	0.0209 (4)	
C24	0.32321 (18)	−0.01977 (12)	0.96786 (12)	0.0265 (5)	
H24	0.3351	−0.0681	0.9869	0.032*	
C25	0.23823 (19)	0.02179 (13)	0.98736 (13)	0.0323 (5)	
H25	0.1907	0.0026	1.0200	0.039*	
C26	0.22311 (18)	0.09185 (13)	0.95879 (13)	0.0322 (5)	
H26	0.1652	0.1215	0.9719	0.039*	
C27	0.29211 (17)	0.11832 (12)	0.91149 (12)	0.0267 (5)	
H27	0.2806	0.1665	0.8919	0.032*	
C28	0.4205 (2)	0.08571 (14)	0.66017 (12)	0.0325 (5)	
H28	0.4873	0.1110	0.6573	0.039*	
C29	0.3602 (2)	0.06282 (16)	0.59510 (13)	0.0414 (6)	
H29	0.3863	0.0708	0.5487	0.050*	
C30	0.2610 (2)	0.02813 (14)	0.59821 (14)	0.0393 (6)	
H30	0.2183	0.0114	0.5542	0.047*	
C31	0.2258 (2)	0.01849 (13)	0.66669 (13)	0.0355 (6)	
H31	0.1568	−0.0034	0.6705	0.043*	
C32	0.29174 (19)	0.04088 (13)	0.72914 (13)	0.0325 (5)	
H32	0.2674	0.0326	0.7761	0.039*	
C33	0.42434 (17)	0.27757 (11)	0.81583 (11)	0.0221 (4)	
C34	0.34423 (19)	0.33600 (12)	0.78780 (13)	0.0303 (5)	
H34A	0.3224	0.3635	0.8299	0.045*	
H34B	0.2796	0.3133	0.7599	0.045*	
H34C	0.3782	0.3695	0.7552	0.045*	
F1	0.19206 (15)	0.25070 (8)	1.03726 (9)	0.0508 (4)	
F2	0.25047 (14)	0.36665 (9)	1.02528 (10)	0.0552 (4)	
F3	0.07127 (14)	0.34422 (11)	1.02327 (11)	0.0668 (5)	
F4	0.08042 (14)	0.26209 (10)	0.93069 (10)	0.0599 (5)	
F5	0.25968 (13)	0.28531 (9)	0.93345 (9)	0.0491 (4)	
F6	0.13802 (14)	0.37771 (9)	0.91763 (10)	0.0571 (5)	
F7	1.08931 (11)	−0.01718 (9)	0.81833 (8)	0.0456 (4)	
F8	1.01735 (13)	0.09573 (8)	0.82953 (9)	0.0482 (4)	
F9	0.96227 (14)	0.00061 (9)	0.89494 (8)	0.0506 (4)	
F10	0.92007 (13)	−0.06315 (8)	0.78882 (9)	0.0507 (4)	
F11	0.97600 (13)	0.03209 (9)	0.72357 (8)	0.0527 (4)	
F12	0.84871 (12)	0.04979 (10)	0.80123 (11)	0.0641 (5)	
N1	0.77176 (13)	0.20484 (9)	0.97898 (9)	0.0206 (4)	
N2	0.60242 (14)	0.27328 (9)	1.01643 (9)	0.0201 (3)	
N3	0.72032 (14)	0.34776 (10)	0.90824 (9)	0.0235 (4)	
N4	0.54145 (13)	0.01119 (9)	0.84978 (9)	0.0203 (4)	
N5	0.37543 (13)	0.07851 (9)	0.89189 (9)	0.0199 (4)	
N6	0.38902 (14)	0.07386 (9)	0.72756 (9)	0.0233 (4)	
O1	0.56711 (11)	0.15277 (7)	0.91364 (7)	0.0193 (3)	
O2	0.70429 (13)	0.22717 (8)	0.81711 (8)	0.0293 (3)	
O3	0.60385 (12)	0.13784 (8)	0.75799 (8)	0.0276 (3)	
O4	0.50483 (12)	0.29827 (8)	0.86021 (8)	0.0280 (3)	
O5	0.40569 (12)	0.21248 (8)	0.79379 (8)	0.0265 (3)	
P1	0.16409 (5)	0.31467 (3)	0.97767 (4)	0.03196 (14)	
P2	0.96918 (5)	0.01609 (4)	0.80911 (3)	0.03143 (14)	
Ru1	0.638351 (13)	0.243804 (9)	0.914219 (9)	0.01857 (5)	
Ru2	0.486442 (13)	0.114865 (9)	0.827033 (9)	0.01832 (5)	

### Atomic displacement parameters (Å^2^)

**Table:**

	*U*^11^	*U*^22^	*U*^33^	*U*^12^	*U*^13^	*U*^23^
C1	0.0253 (11)	0.0236 (11)	0.0326 (12)	−0.0008 (9)	0.0112 (9)	0.0007 (9)
C2	0.0222 (11)	0.0290 (12)	0.0493 (15)	0.0039 (9)	0.0107 (10)	0.0051 (11)
C3	0.0215 (11)	0.0385 (14)	0.0443 (15)	0.0011 (10)	−0.0012 (10)	0.0105 (11)
C4	0.0286 (12)	0.0362 (13)	0.0268 (12)	−0.0015 (10)	−0.0004 (9)	0.0054 (10)
C5	0.0252 (11)	0.0198 (10)	0.0217 (10)	−0.0035 (8)	0.0033 (8)	0.0017 (8)
C6	0.0250 (11)	0.0186 (10)	0.0211 (10)	−0.0027 (8)	0.0025 (8)	0.0008 (8)
C7	0.0354 (12)	0.0278 (12)	0.0216 (11)	−0.0011 (10)	0.0024 (9)	−0.0015 (9)
C8	0.0469 (14)	0.0300 (12)	0.0239 (12)	0.0002 (11)	0.0116 (10)	−0.0057 (9)
C9	0.0316 (12)	0.0300 (12)	0.0344 (13)	0.0028 (10)	0.0162 (10)	−0.0018 (10)
C10	0.0224 (11)	0.0242 (11)	0.0302 (12)	−0.0008 (9)	0.0074 (9)	−0.0010 (9)
C11	0.0326 (12)	0.0295 (12)	0.0290 (12)	−0.0022 (10)	0.0030 (10)	0.0010 (9)
C12	0.0334 (13)	0.0363 (14)	0.0409 (14)	−0.0092 (11)	0.0053 (11)	0.0051 (11)
C13	0.0453 (15)	0.0295 (13)	0.0395 (14)	−0.0119 (11)	−0.0022 (11)	0.0058 (11)
C14	0.0414 (14)	0.0255 (12)	0.0371 (14)	−0.0018 (10)	−0.0061 (11)	0.0020 (10)
C15	0.0293 (12)	0.0262 (11)	0.0327 (12)	−0.0009 (10)	−0.0005 (10)	−0.0018 (9)
C16	0.0272 (11)	0.0219 (10)	0.0194 (10)	0.0006 (9)	0.0067 (8)	0.0023 (8)
C17	0.0424 (14)	0.0391 (14)	0.0280 (12)	−0.0109 (11)	0.0185 (11)	−0.0038 (10)
C18	0.0264 (11)	0.0253 (11)	0.0305 (12)	−0.0015 (9)	0.0099 (9)	−0.0037 (9)
C19	0.0296 (12)	0.0283 (12)	0.0455 (14)	0.0043 (10)	0.0120 (10)	−0.0053 (10)
C20	0.0372 (13)	0.0190 (11)	0.0478 (15)	0.0037 (10)	0.0052 (11)	0.0014 (10)
C21	0.0294 (12)	0.0230 (11)	0.0342 (12)	−0.0030 (9)	0.0037 (10)	0.0038 (9)
C22	0.0225 (10)	0.0205 (10)	0.0193 (10)	−0.0030 (8)	0.0025 (8)	−0.0002 (8)
C23	0.0231 (10)	0.0215 (10)	0.0178 (10)	−0.0043 (8)	0.0020 (8)	−0.0004 (8)
C24	0.0298 (11)	0.0264 (11)	0.0242 (11)	−0.0077 (9)	0.0067 (9)	0.0019 (9)
C25	0.0293 (12)	0.0382 (13)	0.0320 (12)	−0.0111 (10)	0.0144 (10)	−0.0034 (10)
C26	0.0227 (11)	0.0355 (13)	0.0407 (13)	−0.0030 (10)	0.0128 (10)	−0.0070 (10)
C27	0.0240 (11)	0.0239 (11)	0.0323 (12)	0.0000 (9)	0.0043 (9)	−0.0021 (9)
C28	0.0336 (13)	0.0390 (13)	0.0255 (12)	0.0023 (11)	0.0061 (10)	−0.0016 (10)
C29	0.0478 (16)	0.0541 (17)	0.0222 (12)	0.0014 (13)	0.0037 (11)	−0.0051 (11)
C30	0.0478 (15)	0.0375 (14)	0.0297 (13)	0.0008 (12)	−0.0058 (11)	−0.0057 (11)
C31	0.0397 (14)	0.0286 (12)	0.0360 (13)	−0.0075 (11)	−0.0036 (11)	0.0002 (10)
C32	0.0407 (13)	0.0302 (12)	0.0264 (12)	−0.0079 (10)	0.0038 (10)	0.0022 (9)
C33	0.0262 (11)	0.0232 (11)	0.0178 (10)	−0.0007 (9)	0.0056 (8)	0.0016 (8)
C34	0.0361 (13)	0.0245 (11)	0.0289 (12)	0.0042 (10)	−0.0018 (10)	0.0018 (9)
F1	0.0738 (11)	0.0407 (9)	0.0368 (9)	0.0087 (8)	0.0020 (8)	0.0039 (7)
F2	0.0578 (10)	0.0440 (9)	0.0611 (11)	−0.0088 (8)	−0.0035 (8)	−0.0183 (8)
F3	0.0587 (11)	0.0706 (12)	0.0781 (13)	0.0189 (10)	0.0359 (10)	0.0048 (10)
F4	0.0574 (11)	0.0634 (11)	0.0548 (11)	−0.0285 (9)	−0.0091 (9)	0.0039 (8)
F5	0.0485 (9)	0.0500 (9)	0.0522 (9)	0.0037 (8)	0.0190 (7)	−0.0078 (8)
F6	0.0563 (10)	0.0500 (10)	0.0641 (11)	0.0043 (8)	0.0045 (9)	0.0224 (8)
F7	0.0355 (8)	0.0565 (10)	0.0455 (9)	0.0094 (7)	0.0077 (7)	−0.0087 (7)
F8	0.0549 (9)	0.0368 (8)	0.0528 (10)	−0.0076 (7)	0.0061 (8)	−0.0032 (7)
F9	0.0689 (11)	0.0511 (9)	0.0366 (8)	0.0099 (8)	0.0248 (8)	0.0043 (7)
F10	0.0567 (10)	0.0406 (9)	0.0543 (10)	−0.0156 (8)	0.0042 (8)	0.0003 (7)
F11	0.0617 (10)	0.0640 (11)	0.0308 (8)	−0.0206 (9)	−0.0002 (7)	0.0092 (7)
F12	0.0319 (8)	0.0642 (11)	0.0965 (14)	0.0086 (8)	0.0090 (9)	0.0198 (10)
N1	0.0205 (8)	0.0177 (8)	0.0245 (9)	−0.0020 (7)	0.0064 (7)	0.0004 (7)
N2	0.0223 (9)	0.0181 (8)	0.0206 (9)	−0.0015 (7)	0.0049 (7)	−0.0005 (7)
N3	0.0261 (9)	0.0229 (9)	0.0210 (9)	−0.0033 (7)	0.0016 (7)	−0.0016 (7)
N4	0.0222 (9)	0.0179 (8)	0.0210 (9)	−0.0015 (7)	0.0040 (7)	−0.0006 (7)
N5	0.0199 (8)	0.0225 (9)	0.0175 (8)	−0.0026 (7)	0.0026 (7)	−0.0013 (7)
N6	0.0293 (10)	0.0205 (9)	0.0201 (9)	−0.0018 (7)	0.0030 (7)	−0.0004 (7)
O1	0.0195 (7)	0.0200 (7)	0.0191 (7)	−0.0020 (6)	0.0056 (5)	0.0007 (5)
O2	0.0357 (9)	0.0313 (8)	0.0233 (8)	−0.0110 (7)	0.0130 (7)	−0.0049 (6)
O3	0.0336 (8)	0.0309 (8)	0.0204 (7)	−0.0114 (7)	0.0113 (6)	−0.0036 (6)
O4	0.0316 (8)	0.0203 (7)	0.0299 (8)	0.0000 (6)	−0.0048 (7)	0.0001 (6)
O5	0.0320 (8)	0.0199 (8)	0.0258 (8)	0.0002 (6)	−0.0034 (6)	0.0007 (6)
P1	0.0310 (3)	0.0305 (3)	0.0345 (3)	0.0006 (3)	0.0047 (3)	−0.0005 (3)
P2	0.0289 (3)	0.0339 (3)	0.0325 (3)	−0.0013 (3)	0.0077 (2)	0.0024 (3)
Ru1	0.02055 (9)	0.01830 (9)	0.01732 (9)	−0.00145 (6)	0.00417 (6)	−0.00025 (6)
Ru2	0.02204 (9)	0.01740 (9)	0.01607 (8)	−0.00198 (6)	0.00459 (6)	0.00085 (6)

### Geometric parameters (Å, º)

**Table:**

C1—N1	1.340 (3)	C24—C25	1.375 (3)
C1—C2	1.369 (3)	C24—H24	0.9500
C1—H1	0.9500	C25—C26	1.381 (3)
C2—C3	1.377 (4)	C25—H25	0.9500
C2—H2	0.9500	C26—C27	1.370 (3)
C3—C4	1.375 (3)	C26—H26	0.9500
C3—H3	0.9500	C27—N5	1.342 (3)
C4—C5	1.387 (3)	C27—H27	0.9500
C4—H4	0.9500	C28—N6	1.347 (3)
C5—N1	1.367 (3)	C28—C29	1.379 (3)
C5—C6	1.462 (3)	C28—H28	0.9500
C6—N2	1.361 (3)	C29—C30	1.384 (4)
C6—C7	1.387 (3)	C29—H29	0.9500
C7—C8	1.371 (3)	C30—C31	1.379 (4)
C7—H7	0.9500	C30—H30	0.9500
C8—C9	1.382 (3)	C31—C32	1.371 (3)
C8—H8	0.9500	C31—H31	0.9500
C9—C10	1.383 (3)	C32—N6	1.345 (3)
C9—H9	0.9500	C32—H32	0.9500
C10—N2	1.344 (3)	C33—O4	1.255 (2)
C10—H10	0.9500	C33—O5	1.263 (2)
C11—N3	1.337 (3)	C33—C34	1.497 (3)
C11—C12	1.385 (3)	C34—H34A	0.9800
C11—H11	0.9500	C34—H34B	0.9800
C12—C13	1.376 (4)	C34—H34C	0.9800
C12—H12	0.9500	F1—P1	1.5977 (16)
C13—C14	1.379 (4)	F2—P1	1.5956 (16)
C13—H13	0.9500	F3—P1	1.5889 (18)
C14—C15	1.375 (3)	F4—P1	1.5771 (16)
C14—H14	0.9500	F5—P1	1.5997 (16)
C15—N3	1.341 (3)	F6—P1	1.5878 (16)
C15—H15	0.9500	F7—P2	1.5898 (15)
C16—O2	1.250 (2)	F8—P2	1.5936 (16)
C16—O3	1.261 (2)	F9—P2	1.5961 (16)
C16—C17	1.495 (3)	F10—P2	1.5908 (16)
C17—H17A	0.9800	F11—P2	1.5921 (16)
C17—H17B	0.9800	F12—P2	1.5972 (17)
C17—H17C	0.9800	N1—Ru1	2.0258 (17)
C18—N4	1.340 (3)	N2—Ru1	2.0331 (17)
C18—C19	1.382 (3)	N3—Ru1	2.1559 (17)
C18—H18	0.9500	N4—Ru2	2.0316 (17)
C19—C20	1.384 (3)	N5—Ru2	2.0262 (17)
C19—H19	0.9500	N6—Ru2	2.1710 (17)
C20—C21	1.379 (3)	O1—Ru1	1.8762 (13)
C20—H20	0.9500	O1—Ru2	1.8822 (13)
C21—C22	1.385 (3)	O2—Ru1	2.0546 (15)
C21—H21	0.9500	O3—Ru2	2.0737 (14)
C22—N4	1.362 (3)	O4—Ru1	2.0614 (14)
C22—C23	1.463 (3)	O5—Ru2	2.0897 (14)
C23—N5	1.361 (3)	Ru1—Ru2	3.2838 (3)
C23—C24	1.392 (3)		
			
N1—C1—C2	122.2 (2)	N6—C32—C31	123.6 (2)
N1—C1—H1	118.9	N6—C32—H32	118.2
C2—C1—H1	118.9	C31—C32—H32	118.2
C1—C2—C3	119.3 (2)	O4—C33—O5	125.66 (19)
C1—C2—H2	120.4	O4—C33—C34	116.05 (18)
C3—C2—H2	120.4	O5—C33—C34	118.29 (18)
C4—C3—C2	119.5 (2)	C33—C34—H34A	109.5
C4—C3—H3	120.3	C33—C34—H34B	109.5
C2—C3—H3	120.3	H34A—C34—H34B	109.5
C3—C4—C5	119.2 (2)	C33—C34—H34C	109.5
C3—C4—H4	120.4	H34A—C34—H34C	109.5
C5—C4—H4	120.4	H34B—C34—H34C	109.5
N1—C5—C4	120.7 (2)	C1—N1—C5	118.87 (18)
N1—C5—C6	114.60 (17)	C1—N1—Ru1	126.35 (15)
C4—C5—C6	124.6 (2)	C5—N1—Ru1	114.73 (13)
N2—C6—C7	120.8 (2)	C10—N2—C6	118.92 (18)
N2—C6—C5	114.47 (18)	C10—N2—Ru1	125.98 (14)
C7—C6—C5	124.62 (19)	C6—N2—Ru1	114.86 (13)
C8—C7—C6	119.9 (2)	C11—N3—C15	117.61 (19)
C8—C7—H7	120.1	C11—N3—Ru1	121.24 (15)
C6—C7—H7	120.1	C15—N3—Ru1	120.82 (15)
C7—C8—C9	119.3 (2)	C18—N4—C22	119.15 (18)
C7—C8—H8	120.4	C18—N4—Ru2	124.77 (15)
C9—C8—H8	120.4	C22—N4—Ru2	116.05 (13)
C8—C9—C10	118.9 (2)	C27—N5—C23	118.73 (18)
C8—C9—H9	120.5	C27—N5—Ru2	125.00 (15)
C10—C9—H9	120.5	C23—N5—Ru2	116.19 (13)
N2—C10—C9	122.1 (2)	C32—N6—C28	116.84 (19)
N2—C10—H10	118.9	C32—N6—Ru2	122.50 (15)
C9—C10—H10	118.9	C28—N6—Ru2	120.48 (15)
N3—C11—C12	122.7 (2)	Ru1—O1—Ru2	121.79 (7)
N3—C11—H11	118.7	C16—O2—Ru1	132.48 (14)
C12—C11—H11	118.7	C16—O3—Ru2	131.07 (13)
C13—C12—C11	119.1 (2)	C33—O4—Ru1	132.87 (14)
C13—C12—H12	120.4	C33—O5—Ru2	130.27 (13)
C11—C12—H12	120.4	F4—P1—F6	90.36 (10)
C12—C13—C14	118.5 (2)	F4—P1—F3	91.27 (11)
C12—C13—H13	120.7	F6—P1—F3	90.89 (10)
C14—C13—H13	120.7	F4—P1—F2	178.90 (11)
C15—C14—C13	119.2 (2)	F6—P1—F2	90.34 (10)
C15—C14—H14	120.4	F3—P1—F2	89.57 (10)
C13—C14—H14	120.4	F4—P1—F1	89.63 (9)
N3—C15—C14	122.9 (2)	F6—P1—F1	178.96 (11)
N3—C15—H15	118.5	F3—P1—F1	90.15 (10)
C14—C15—H15	118.5	F2—P1—F1	89.66 (9)
O2—C16—O3	125.82 (19)	F4—P1—F5	89.94 (10)
O2—C16—C17	116.58 (19)	F6—P1—F5	89.76 (10)
O3—C16—C17	117.60 (19)	F3—P1—F5	178.62 (11)
C16—C17—H17A	109.5	F2—P1—F5	89.21 (9)
C16—C17—H17B	109.5	F1—P1—F5	89.19 (9)
H17A—C17—H17B	109.5	F7—P2—F10	90.05 (9)
C16—C17—H17C	109.5	F7—P2—F11	90.53 (9)
H17A—C17—H17C	109.5	F10—P2—F11	90.34 (9)
H17B—C17—H17C	109.5	F7—P2—F8	90.43 (9)
N4—C18—C19	122.1 (2)	F10—P2—F8	179.50 (10)
N4—C18—H18	119.0	F11—P2—F8	89.78 (9)
C19—C18—H18	119.0	F7—P2—F9	89.64 (9)
C18—C19—C20	118.9 (2)	F10—P2—F9	89.99 (9)
C18—C19—H19	120.5	F11—P2—F9	179.63 (10)
C20—C19—H19	120.6	F8—P2—F9	89.90 (9)
C21—C20—C19	119.5 (2)	F7—P2—F12	179.08 (10)
C21—C20—H20	120.3	F10—P2—F12	90.31 (10)
C19—C20—H20	120.3	F11—P2—F12	90.31 (10)
C20—C21—C22	119.3 (2)	F8—P2—F12	89.20 (10)
C20—C21—H21	120.4	F9—P2—F12	89.52 (10)
C22—C21—H21	120.4	O1—Ru1—N1	92.23 (6)
N4—C22—C21	121.12 (19)	O1—Ru1—N2	94.74 (6)
N4—C22—C23	114.24 (17)	N1—Ru1—N2	79.50 (7)
C21—C22—C23	124.6 (2)	O1—Ru1—O2	95.78 (6)
N5—C23—C24	121.07 (19)	N1—Ru1—O2	93.76 (7)
N5—C23—C22	114.34 (18)	N2—Ru1—O2	167.72 (6)
C24—C23—C22	124.59 (19)	O1—Ru1—O4	94.44 (6)
C25—C24—C23	119.4 (2)	N1—Ru1—O4	170.41 (6)
C25—C24—H24	120.3	N2—Ru1—O4	93.07 (6)
C23—C24—H24	120.3	O2—Ru1—O4	92.41 (6)
C24—C25—C26	118.9 (2)	O1—Ru1—N3	176.78 (6)
C24—C25—H25	120.6	N1—Ru1—N3	89.16 (7)
C26—C25—H25	120.6	N2—Ru1—N3	88.37 (7)
C27—C26—C25	119.7 (2)	O2—Ru1—N3	81.23 (6)
C27—C26—H26	120.2	O4—Ru1—N3	84.53 (6)
C25—C26—H26	120.2	O1—Ru2—N5	87.68 (6)
N5—C27—C26	122.2 (2)	O1—Ru2—N4	92.50 (6)
N5—C27—H27	118.9	N5—Ru2—N4	79.09 (7)
C26—C27—H27	118.9	O1—Ru2—O3	95.44 (6)
N6—C28—C29	122.9 (2)	N5—Ru2—O3	172.56 (6)
N6—C28—H28	118.6	N4—Ru2—O3	94.00 (7)
C29—C28—H28	118.6	O1—Ru2—O5	96.39 (6)
C28—C29—C30	119.2 (2)	N5—Ru2—O5	96.52 (6)
C28—C29—H29	120.4	N4—Ru2—O5	169.94 (6)
C30—C29—H29	120.4	O3—Ru2—O5	89.85 (6)
C31—C30—C29	118.4 (2)	O1—Ru2—N6	178.03 (6)
C31—C30—H30	120.8	N5—Ru2—N6	91.55 (7)
C29—C30—H30	120.8	N4—Ru2—N6	89.12 (6)
C32—C31—C30	119.0 (2)	O3—Ru2—N6	85.54 (6)
C32—C31—H31	120.5	O5—Ru2—N6	81.90 (6)
C30—C31—H31	120.5		
			
N1—C1—C2—C3	−3.5 (3)	Ru2—O1—Ru1—N2	141.42 (9)
C1—C2—C3—C4	3.6 (4)	Ru2—O1—Ru1—O2	−44.92 (9)
C2—C3—C4—C5	0.3 (4)	Ru2—O1—Ru1—O4	47.96 (9)
C3—C4—C5—N1	−4.5 (3)	C1—N1—Ru1—O1	76.40 (17)
C3—C4—C5—C6	172.0 (2)	C5—N1—Ru1—O1	−106.27 (14)
N1—C5—C6—N2	−0.6 (3)	C1—N1—Ru1—N2	170.82 (18)
C4—C5—C6—N2	−177.2 (2)	C5—N1—Ru1—N2	−11.86 (14)
N1—C5—C6—C7	176.1 (2)	C1—N1—Ru1—O2	−19.53 (17)
C4—C5—C6—C7	−0.5 (3)	C5—N1—Ru1—O2	157.79 (14)
N2—C6—C7—C8	0.9 (3)	C1—N1—Ru1—N3	−100.69 (17)
C5—C6—C7—C8	−175.7 (2)	C5—N1—Ru1—N3	76.63 (14)
C6—C7—C8—C9	0.0 (3)	C10—N2—Ru1—O1	−82.78 (17)
C7—C8—C9—C10	−0.8 (3)	C6—N2—Ru1—O1	102.95 (14)
C8—C9—C10—N2	0.7 (3)	C10—N2—Ru1—N1	−174.18 (18)
N3—C11—C12—C13	−0.2 (4)	C6—N2—Ru1—N1	11.56 (14)
C11—C12—C13—C14	−0.4 (4)	C10—N2—Ru1—O2	128.4 (3)
C12—C13—C14—C15	0.4 (4)	C6—N2—Ru1—O2	−45.9 (4)
C13—C14—C15—N3	0.3 (4)	C10—N2—Ru1—O4	11.93 (17)
N4—C18—C19—C20	−0.3 (4)	C6—N2—Ru1—O4	−162.33 (14)
C18—C19—C20—C21	0.5 (4)	C10—N2—Ru1—N3	96.37 (17)
C19—C20—C21—C22	0.4 (3)	C6—N2—Ru1—N3	−77.89 (14)
C20—C21—C22—N4	−1.7 (3)	C16—O2—Ru1—O1	18.3 (2)
C20—C21—C22—C23	178.3 (2)	C16—O2—Ru1—N1	110.9 (2)
N4—C22—C23—N5	2.3 (2)	C16—O2—Ru1—N2	167.1 (3)
C21—C22—C23—N5	−177.67 (19)	C16—O2—Ru1—O4	−76.4 (2)
N4—C22—C23—C24	−178.25 (19)	C16—O2—Ru1—N3	−160.5 (2)
C21—C22—C23—C24	1.8 (3)	C33—O4—Ru1—O1	−26.5 (2)
N5—C23—C24—C25	−0.3 (3)	C33—O4—Ru1—N2	−121.5 (2)
C22—C23—C24—C25	−179.77 (19)	C33—O4—Ru1—O2	69.46 (19)
C23—C24—C25—C26	−0.1 (3)	C33—O4—Ru1—N3	150.4 (2)
C24—C25—C26—C27	0.4 (3)	C11—N3—Ru1—N1	43.68 (17)
C25—C26—C27—N5	−0.4 (3)	C15—N3—Ru1—N1	−143.18 (16)
N6—C28—C29—C30	2.1 (4)	C11—N3—Ru1—N2	123.20 (17)
C28—C29—C30—C31	0.7 (4)	C15—N3—Ru1—N2	−63.66 (16)
C29—C30—C31—C32	−2.5 (4)	C11—N3—Ru1—O2	−50.25 (16)
C30—C31—C32—N6	1.8 (4)	C15—N3—Ru1—O2	122.88 (16)
C2—C1—N1—C5	−0.6 (3)	C11—N3—Ru1—O4	−143.55 (17)
C2—C1—N1—Ru1	176.58 (16)	C15—N3—Ru1—O4	29.58 (16)
C4—C5—N1—C1	4.7 (3)	Ru1—O1—Ru2—N5	−140.72 (9)
C6—C5—N1—C1	−172.11 (18)	Ru1—O1—Ru2—N4	140.31 (9)
C4—C5—N1—Ru1	−172.88 (16)	Ru1—O1—Ru2—O3	46.05 (9)
C6—C5—N1—Ru1	10.4 (2)	Ru1—O1—Ru2—O5	−44.41 (9)
C9—C10—N2—C6	0.2 (3)	C27—N5—Ru2—O1	86.17 (16)
C9—C10—N2—Ru1	−173.88 (16)	C23—N5—Ru2—O1	−90.44 (14)
C7—C6—N2—C10	−1.0 (3)	C27—N5—Ru2—N4	179.16 (17)
C5—C6—N2—C10	175.91 (18)	C23—N5—Ru2—N4	2.56 (13)
C7—C6—N2—Ru1	173.72 (16)	C27—N5—Ru2—O5	−10.00 (17)
C5—C6—N2—Ru1	−9.4 (2)	C23—N5—Ru2—O5	173.39 (13)
C12—C11—N3—C15	0.8 (3)	C27—N5—Ru2—N6	−92.03 (17)
C12—C11—N3—Ru1	174.11 (17)	C23—N5—Ru2—N6	91.37 (14)
C14—C15—N3—C11	−0.8 (3)	C18—N4—Ru2—O1	−96.37 (16)
C14—C15—N3—Ru1	−174.20 (17)	C22—N4—Ru2—O1	85.88 (14)
C19—C18—N4—C22	−1.0 (3)	C18—N4—Ru2—N5	176.48 (17)
C19—C18—N4—Ru2	−178.64 (16)	C22—N4—Ru2—N5	−1.26 (14)
C21—C22—N4—C18	1.9 (3)	C18—N4—Ru2—O3	−0.73 (17)
C23—C22—N4—C18	−178.00 (17)	C22—N4—Ru2—O3	−178.48 (14)
C21—C22—N4—Ru2	179.81 (15)	C18—N4—Ru2—O5	111.5 (4)
C23—C22—N4—Ru2	−0.1 (2)	C22—N4—Ru2—O5	−66.2 (4)
C26—C27—N5—C23	0.1 (3)	C18—N4—Ru2—N6	84.74 (17)
C26—C27—N5—Ru2	−176.45 (16)	C22—N4—Ru2—N6	−93.01 (14)
C24—C23—N5—C27	0.3 (3)	C16—O3—Ru2—O1	−21.95 (19)
C22—C23—N5—C27	179.81 (17)	C16—O3—Ru2—N4	−114.85 (18)
C24—C23—N5—Ru2	177.12 (15)	C16—O3—Ru2—O5	74.45 (18)
C22—C23—N5—Ru2	−3.4 (2)	C16—O3—Ru2—N6	156.35 (19)
C31—C32—N6—C28	0.9 (3)	C33—O5—Ru2—O1	13.71 (19)
C31—C32—N6—Ru2	175.96 (18)	C33—O5—Ru2—N5	102.09 (18)
C29—C28—N6—C32	−2.9 (3)	C33—O5—Ru2—N4	165.6 (3)
C29—C28—N6—Ru2	−178.03 (19)	C33—O5—Ru2—O3	−81.75 (18)
O3—C16—O2—Ru1	3.0 (3)	C33—O5—Ru2—N6	−167.27 (19)
C17—C16—O2—Ru1	−177.00 (15)	C32—N6—Ru2—N5	1.68 (17)
O2—C16—O3—Ru2	−0.7 (3)	C28—N6—Ru2—N5	176.54 (17)
C17—C16—O3—Ru2	179.31 (15)	C32—N6—Ru2—N4	80.75 (17)
O5—C33—O4—Ru1	1.3 (3)	C28—N6—Ru2—N4	−104.39 (17)
C34—C33—O4—Ru1	−179.14 (15)	C32—N6—Ru2—O3	174.83 (18)
O4—C33—O5—Ru2	6.5 (3)	C28—N6—Ru2—O3	−10.31 (17)
C34—C33—O5—Ru2	−173.03 (14)	C32—N6—Ru2—O5	−94.69 (17)
Ru2—O1—Ru1—N1	−138.93 (9)	C28—N6—Ru2—O5	80.17 (17)

### Hydrogen-bond geometry (Å, º)

**Table:**

*D*—H···*A*	*D*—H	H···*A*	*D*···*A*	*D*—H···*A*
C9—H9···F2	0.95	2.53	3.227 (3)	130
C10—H10···F5	0.95	2.48	3.362 (3)	154
C10—H10···O4	0.95	2.59	3.105 (3)	115
C12—H12···F6^i^	0.95	2.42	3.270 (3)	149
C15—H15···O4	0.95	2.43	2.904 (3)	110
C17—H17*A*···F1^ii^	0.98	2.34	3.240 (3)	153
C18—H18···F12	0.95	2.37	3.090 (3)	132
C18—H18···O3	0.95	2.52	3.096 (3)	119
C24—H24···O1^iii^	0.95	2.56	3.405 (3)	149
C27—H27···F5	0.95	2.32	3.101 (3)	139
C28—H28···O3	0.95	2.23	2.855 (3)	122
C32—H32···N5	0.95	2.49	3.080 (3)	121
C34—H34*C*···F7^iv^	0.98	2.52	3.455 (3)	160

Symmetry codes: (i) *x*+1, *y*, *z*; (ii) *x*+1/2, −*y*+1/2, *z*−1/2; (iii) −*x*+1, −*y*, −*z*+2; (iv) −*x*+3/2, *y*+1/2, −*z*+3/2.
